# Quality of Life Predictors in a Group of Informal Caregivers during the COVID-19 Pandemic

**DOI:** 10.3390/medicina59081486

**Published:** 2023-08-18

**Authors:** Ana Claudia Damian, Alexandra Ioana Mihăilescu, Cristina Anghele, Constantin Alexandru Ciobanu, Cristian Petrescu, Sorin Riga, Vlad Dionisie, Adela Magdalena Ciobanu

**Affiliations:** 1 Neuroscience Department, Discipline of Psychiatry, Faculty of Medicine, “Carol Davila” University of Medicine and Pharmacy, 020021 Bucharest, Romania; clau_damian@yahoo.com (A.C.D.);cristian.petrescu@drd.umfcd.ro (C.P.); adela.ciobanu@yahoo.com (A.M.C.); 2 Department of Psychiatry, ‘Prof. Dr. Alexandru Obregia’ Clinical Hospital of Psychiatry, 041914 Bucharest, Romania; alexandra.mihailescu@umfcd.ro (A.I.M.); vlad.dionisie@gmail.com (V.D.); 3 Department of Medical Psychology, Faculty of Medicine, University of Medicine and Pharmacy “Carol Davila”, 050474 Bucharest, Romania; 4 Faculty of Medicine, “Titu Maiorescu” University of Medicine and Pharmacy, 040441 Bucharest, Romania; 5 Department of Stress Research and Prophylaxis, ‘Prof. Dr. Alexandru Obregia’ Clinical Hospital of Psychiatry, 041914 Bucharest, Romania; d_s_riga@yahoo.com; 6 Romanian Academy of Medical Sciences, 927180 Bucharest, Romania; 7 Department of Psychiatry and Psychology, “Carol Davila” University of Medicine and Pharmacy, 020021 Bucharest, Romania

**Keywords:** informal caregiver, burnout, anxiety, depression, life quality

## Abstract

*Background and Objectives*: The informal caregiver’s contribution to the wellbeing of dementia patients is critical since these individuals become dependent on others for all daily activities. Our goal was to investigate the dynamics of anxiety, depression, burnout, sleep, and their influence on quality of life over a 6-month period in the context of pandemic distress in a sample of informal caregivers of Alzheimer’s patients. *Materials and Methods*: For this prospective, longitudinal study, we conducted a 6-month telephonic survey between 2021 and 2022, administering a series of questionnaires at three timepoints (baseline, 3 months and 6 months) to a group of informal caregivers of patients suffering from dementia due to Alzheimer’s disease. *Results*: A total of 110 caregivers were included at baseline, out of which 96 continued to the second stage and 78 followed through to the last stage. The majority of the participants were female (most likely the patients’ daughters), around 55 years old, living in urban areas, married, with children, having a high school degree or a higher education degree, and working in jobs that required physical presence; in the best-case scenario, they were sharing their responsibilities with another two–three caregivers. More than half of the 110 participants (50.9%) reported mild to moderate anxiety at baseline, and 27.3% reported significant anxiety, with no changes between the three timepoints, F(2, 154) = 0.551, *p* = 0.57; 25% reported moderate–severe depression at the start, with no changes between the three timepoints, F(2, 154) = 2.738, *p* = 0.068; and many reported a decrease in quality of life, poor quality of sleep, and decreased fear of COVID infection. Cynicism, professional effectiveness, anxiety, depression, and sleep quality explained up to 87.8% of the variance in quality of life. *Conclusions*: Caregivers’ decreased quality of life during the pandemic was explained by their levels of burnout, anxiety, and depression throughout the 6-month period.

## 1. Introduction

Formerly known as dementia, neurocognitive disorder causes disabilities in daily activities, rendering patients dependent on their caregivers, as 10% of adults over 65 years and 50% of adults over 90 years are diagnosed with dementia. Worldwide, over 25 million people are diagnosed with dementia, and it is estimated that these numbers will double in the next 20 years. Each year, there are over 10 million new cases of dementia globally, with a mean life expectancy after the diagnosis of 4.5 years [1].

In Romania, the lack of a national register is a major impediment to the development of institutional structure. However, more than 270,000 people are thought to have dementia, with only 35,000 receiving a diagnosis [2].

This condition is distinguished by a progressive deterioration in cognitive function, mood and sleep pattern changes, hostility, miscommunication, hallucinations, and delusions. In their later stages, patients suffering from dementia require assistance with everyday tasks (i.e., walking, eating, taking medicines, bathing), as well as constant observation [3].

During the course of the disease, caregivers frequently serve as advocates and care coordinators, assisting with appointment scheduling, healthcare-system connections, interacting with physicians and ordering prescriptions, emotional support and companionship, feeding, grooming, shopping, money management, and transportation [2]. In their later stages, patients suffering from dementia require assistance with everyday tasks (i.e., walking, eating, taking medicines, bathing), as well as constant observation. Importantly, the caregiver’s role becomes more demanding while the disease progresses, lowering their quality of life [4].

The name “informal” stands for caregivers that have no training and receive no money in exchange for their help; therefore, they have no realistic expectation for the course of the disease, making this a stressful experience even without the COVID-19 pandemic. Other negative aspects include the financial strain, difficulty navigating their caregiver’s role, lack of support, and mental health struggles (anxiety and depression). However, this experience comes with positive aspects also, such as feelings of reciprocity and spiritual wellbeing, since most carers are family members or friends [5,6].

Additional risk factors for a lower quality of life are female gender, close relationship with the patient (often wife/husband), stressful situations, poor physical health, family history of psychiatric diseases, the quality of the relationship with the patient, low self-esteem, and the patient’s behavioral and psychological symptoms [7,8].

The global pandemic of coronavirus disease-19 (COVID-19) has negatively affected the evolution of the disease in the case of dementia patients. Firstly, it has done so by placing these vulnerable elderly individuals with potential comorbidities at risk for acute respiratory infections. Secondly, it has had negative effects by altering their daily routine and imposing new rules (e.g., wearing protective masks, frequent use of hand sanitizer, social isolation, and regular COVID testing) on already low-compliant patients [9,10].

Additionally, during the pandemic, the burden of caregivers was increased due to fear of the virus, uncertainty, lack of support, feelings of isolation, the need to sacrifice work-related obligations, rapid progression of dementia following COVID infection, reduced access to healthcare facilities or formal care services, and the discontinuation of formal caregiving [11].

In Germany, for example, provision of ambulant care was affected by a staff shortage, care centers had to be closed, rehabilitation centers and even hospitals sent patients home to free up capacity for COVID-19 patients, and migrant workers providing care returned home [12,13,14]. As a result, caregivers reported a deterioration of the care situation following the pandemic, with increased burdens, trouble sleeping, worsening mental and physical health, and social isolation [15,16,17].

Thus, the issue of the quality of life of carers for dementia patients is complex and impacted by a variety of circumstances. Clearly, the coronavirus pandemic has had a global impact on mental health and quality of life; however, because there are no public databases that have tracked the incidence and frequency of mental health issues during the pandemic in Romania, we do not know what we are up against. As a result, our goal was to investigate the dynamics of anxiety, depression, burnout, sleep, and quality of life over 6 months in the context of pandemic distress, and the factors that could influence quality of life during this time, in a sample of informal caregivers of patients with dementia.

## 2. Materials and Methods

### 2.1. Study Design

For this prospective and longitudinal study, we conducted a telephonic survey over the course of 6 months between 2021 and 2022, applying a set of questionnaires to a group of informal carers of dementia patients admitted between 2020 and 2021 to “Prof. Dr. Al. Obregia” Psychiatry Hospital in Romania. This study has been authorized by the Ethics Committee of the “Prof. Dr. Alexandru Obregia” Psychiatry Hospital (approval number 73/7 October 2021).

The study inclusion and data collection period overlapped with Romania’s partial lockdown due to the coronavirus pandemic and the vaccination process. Romania endured the fourth wave, the most severe of all infection waves, during this time period, with the second lowest proportion of completely vaccinated people, exposing weak vaccine education, misinformation campaigns, and distrust in the state institutions. Despite the severity of the wave and the pressure on the healthcare system, the government chose a more relaxed approach, imposing a digital green certificate for daily activities [18].

The Ethics Committee from “Prof. Dr. Alexandru Obregia” Psychiatry Hospital approved this research (approval no 73/7 October 2021). All participants included in the study provided written informed consent after the procedures of the study had been fully explained, in accordance with the Declaration of Helsinki and the country’s law.

**Figure 1:**
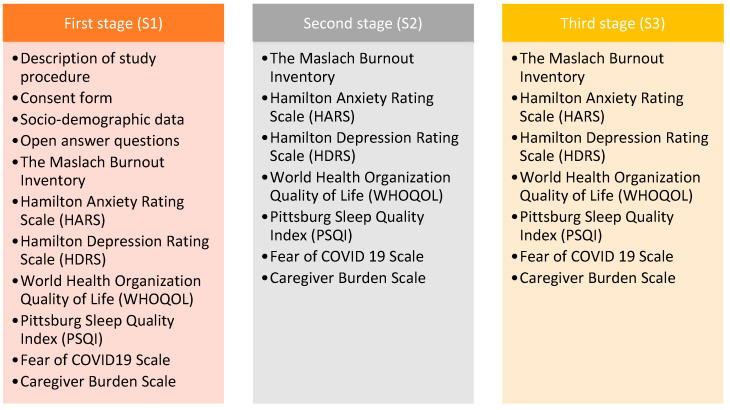
Diagram of the study procedure.

The participants were evaluated at three timepoints over the course of six months: the first stage (S1) was at their inclusion in the study; the second stage (S2) was three months after the baseline assessment; the third stage (S3) was six months from baseline. At baseline (S1), each participant provided sociodemographic and clinical data regarding themselves and the patient they cared for and responded to two questions concerning their difficulties experienced as an informal caregiver and the support they wished they had been offered. Additionally, at all timepoints, participants responded to questionnaires regarding anxiety and depressive symptoms, burnout, quality of life (QoL), sleep quality, caregiver burden, and fear of COVID-19. All subjects were assessed individually over the phone, as in Figure 1.

### 2.2. Measures

The assessment of informal caregivers consisted of a series of questionnaires (Table 1), using the validated Romanian version, as follows:

- Socio-demographic data: we designed a semi-structured questionnaire to collect the following information: gender, age, living area, marital status, children, education, profession, working status. It included COVID questions regarding infection and vaccination. There were questions relating to the patient: relationship to the caregiver, diagnosis, last Mini Mental State Examination (MMSE) score, the number of caregivers for the same patient [19].
- Open-answer questions: “What difficulties have you encountered while being an informal caregiver?” and “What type of assistance do you wish you had?”
- The Maslach Burnout Inventory—General Survey: considered a “gold standard” method for measuring burnout using a 16-item scale with a 7-point Likert-type scale that addresses the following components: exhaustion (Cronbach’s alpha coefficient rating: 0.90), cynicism (Cronbach’s alpha coefficient rating: 0.76), and professional efficacy (Cronbach’s alpha coefficient rating: 0.76) (e.g., “I feel emotionally drained from my work”). For test–retest reliability, time periods of a couple of weeks (scores of 20.60–0.82), 3 months, and 1 year (scores of 0.54–0.60) were used. Score interpretation for exhaustion was 0–18 for low burnout level, 19–26 for moderate, ≥27 for high; for cynicism, 1–5 was a low level of burnout, 6–9 was moderate, ≥10 was high; for professional efficacy, ≥40 was a low level of burnout, 34–39 was moderate, 0–33 was high [20,21].
- The Hamilton Anxiety Rating Scale (HARS): designed for measuring the severity of anxiety through 14 items for both psychic and somatic symptoms (e.g., “Anxious mood: Worries, anticipation of the worst, fearful anticipation, irritability”); the scale is clinician-administered, and a score of ≤17 indicates mild anxiety, 18–24 moderate anxiety, and 25–30 moderate to severe anxiety. The predictive validity using Cronbach’s alpha coefficient was 0.921 [22].
- Hamilton Depression Rating Scale (HDRS): designed for measuring the severity of depression, with 17 items describing both psychic and somatic symptoms (e.g., “Depressed mood: Gloomy attitude, pessimism about the future, feeling of sadness, tendency to weep”); the scale is clinician-administered, and a score of 0–7 is considered normal, 8–16 is mild depression, 17–23 is moderate depression, and ≥24 is severe depression. This scale has adequate internal consistency (α = 0.77), a high degree of inter-rater reliability (ICC = 0.82), and a Cronbach’s alpha coefficient <0.01 [23].
- World Health Organization Quality of Life (WHOQOL) brief version: used for measuring occupational burnout through 26 items assessing four areas: firstly, physical health (seven questions regarding daily activities, mobility, energy, functional capacity, pain, and sleep); secondly, mental health (six questions related to self-image, ability to learn, positive attitudes, negative thoughts, mentality, religion, focus, and mental status); thirdly, social relationships (three questions about personal relationships, sex life, social support); and lastly, environmental health (eight questions about safety, financial resources, health and social services, the environment in which they live, recreation, opportunities to acquire new knowledge, the environment, and their means of transport), e.g., “To what extent do you have the opportunity for leisure activities?” The World Health Organization developed this widely used scale for cross-cultural comparison. The reliability is assessed using the Cronbach’s alpha coefficient (0.91) and, according to the convergent validity, the correlation coefficient values are strongly associated at 0.01 [24,25].
- Pittsburg Sleep Quality Index (PSQI): evaluates the overall sleep quality in the prior month (sleep quality, latency, duration, efficiency, sleep disturbances, the use of sleep medication, and dysfunction during the daytime), e.g., “During the past month, how would you rate your sleep quality overall?” The validity of this test using Cronbach’s alpha coefficient is between 0.70 and 0.85 [26].
- Fear of COVID-19 scale: was developed by a group of researchers in Iran for measuring fear of COVID using 7 questions with 5 possible answers (e.g., “I cannot sleep because I am worried about getting COVID-19”). Total scores range between 7 and 35. This scale has been shown to be a valid and reliable way to assess the fear of COVID in the general population, with robust psychometric properties (test–retest reliability ICC = 0.72, internal consistency of α = 0.82) [27,28].
- Caregiver burden scale: used for measuring the burden of the caregivers using 15 items describing the assistance provided to the patient: transportation, housekeeping, cooking, shopping, decision making, financial record keeping, walking, making house repairs, farming/yardwork, administering medication, dressing, bathing, eating, toileting, and leaving patient unattended (e.g., “Does the patient need assistance with transportation?”), with a total score range between 0 and 15.

### 2.3. Participants

Participants were included in the study if they met specific inclusion and exclusion criteria. The following inclusion criteria were applied: 1. the participant had the role of an informal caregiver of a patient admitted to “Prof. Dr. Al. Obregia” Psychiatry Hospital in Bucharest, Romania; 2. the participant agreed to participate in the study and signed the informed consent; 3. the participant was an informal caregiver of a patient diagnosed with any type of dementia except for Lewy body dementia; 4. the participant had no substance abuse 12 months prior to study enrolment. The following exclusion criteria were applied: 1. formal caregivers; 2. informal caregivers of patient diagnosed with Lewy body dementia; 3. participants were diagnosed with any psychiatric illness or had a history of substance abuse in the last 12 months prior to the study enrolment.

**Figure 2:**
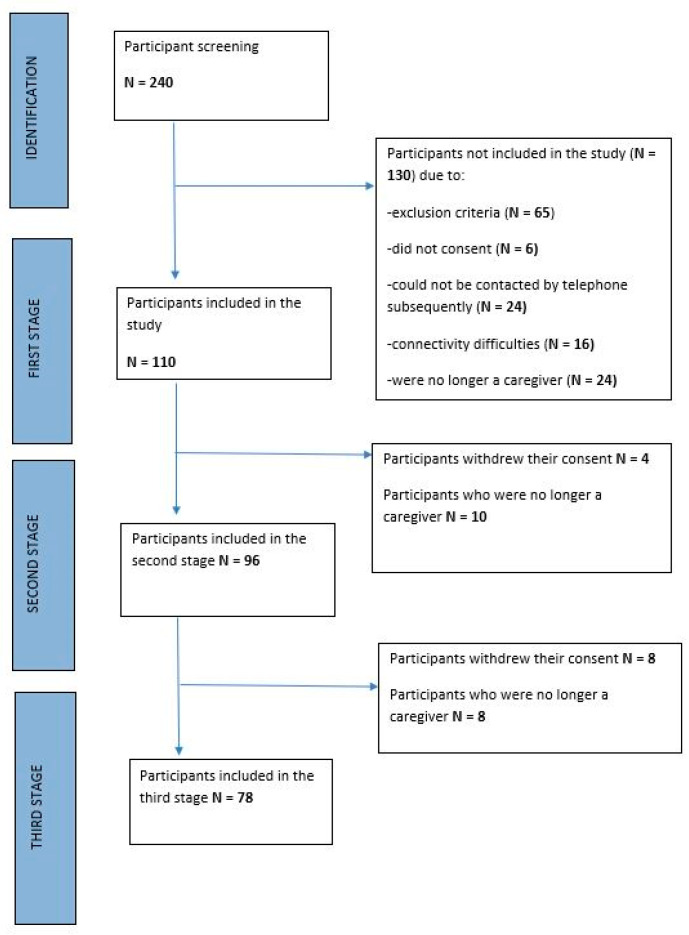
Flowchart of the study population.

For this research, 110 participants were included at baseline, as shown in the flowchart of the study population (Figure 2).

### 2.4. Statistical Analysis

The IBM Statistical Package for Social Sciences (SPSS) version 20 software for Windows was used for statistical analysis. Mean and standard deviation were used for quantitative data, as well as the Pearson correlations, and the frequency and percentage were used for qualitative data. For interpretation of the results, we selected several *t*-tests and ANOVA (analysis of variance), and linear regressions were employed in order to analyze the predictor variables. The stepwise forward approach was used to create multivariate linear regression models. The quality-of-life score was the dependent variable. Professional efficacy, cynicism, exhaustion, anxiety, depression, sleep quality, and caregiver burden were the variables tested for independent predictive ability. A *p* level < 0.05 was considered statistically significant.

## 3. Results

### 3.1. Socio-Demographic Data

A total of 110 caregivers responded to our questionnaire, most of them female (N = 78, 70.9%), with a mean age of 55.2 years old, living in an urban area (N = 88, 80%), married (N = 76, 69.1%), with a high school education (N = 58, 52.7%), employed (N = 68, 61.8%), and with a job requiring physical presence (N = 48, 43.6%), see Table 2.

### 3.2. Questions Regarding the Dementia Patient

Each patient had between one and four informal caregivers, most of them being their son or daughter (N = 68, 61.8), followed by their wife or their husband (N = 20, 18.2). More than half of the patients were diagnosed with a mixed form of dementia (N = 64, 58.2), and they had a mean MMSE score of 13.32 out of 30. The caregiver burden scale was also included in this part since it illustrates how much aid the carers provide to the patient. In this case, the mean score was 11 (See Table 3).

### 3.3. COVID-Related Questions

Our caregivers had a high rate of vaccination (N = 80, 72.7%) correlated with a low rate of infection (N = 22, 20%), while the patients had a frequency of vaccination of 52 (47.3%) and a low rate of infectivity (N = 8, 7.3%).

### 3.4. Caregivers’ Burden and Needs

**Figure 3:**
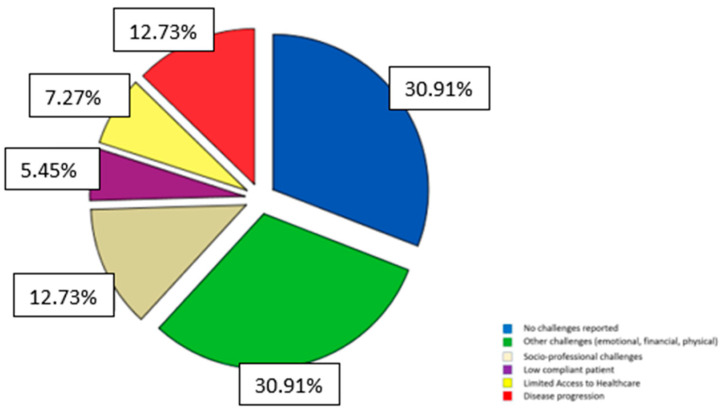
Caregivers’ challenges during COVID.

During COVID, 30.9% of informal carers indicated numerous problems (emotional, financial, and physical). More than a quarter of the individuals reported socio-professional issues (12.73%) or illness progression (12.73%). Finally, some of them mentioned a lack of access to the healthcare system (7.27%) and a lack of patient compliance with environmental issues (5.45%) (See Figure 3).

**Figure 4:**
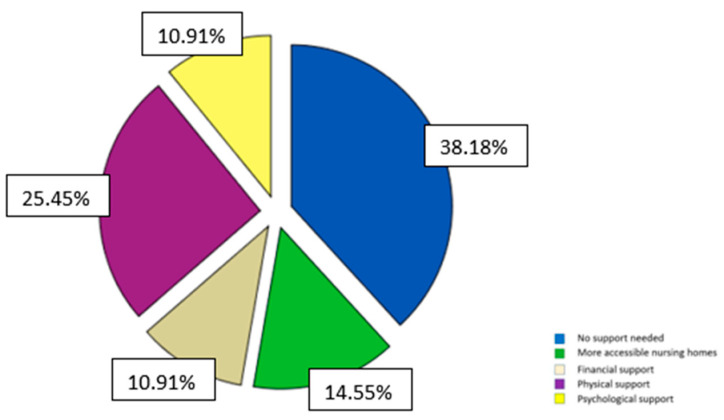
Caregivers’ needs during COVID.

When asked about their requirements, the most often expressed were physical help (25.45%), more accessible nursing homes (14.55%), and financial (10.91%) and psychological support (10.91%) (See Figure 4).

### 3.5. Questionnaires

A total of 110 carers were included in the first stage. The vast majority of participants reported low levels of exhaustion (N = 104, 95.5%), low levels of cynicism (N = 70, 63.6%), high levels of professional efficacy (N = 108, 98.2%), mild anxiety (N = 38, 34.5%), moderate–severe anxiety (N = 30, 27.3%), no depressive symptoms (N = 60, 54.5%), severe depression (N = 28, 25.5%), and a satisfactory level of sleep quality (N = 84, 76.4%), with a mean score of quality of life of 58.71.

The 96 participants included in the second stage had a low exhaustion rate (N = 84, 76.4%), a low level of cynicism (N = 42, 38.2%), a high degree of professional efficacy (N = 96, 87.3%), and a modest level of anxiety (N = 32, 29.1%) with some members experiencing severe anxiety (N = 30, 27.3%), and less than half of the participants reported no depressive symptoms (N = 50, 45.5%) and a satisfactory sleep quality (N = 68, 61.8%), with a mean score of quality of life of 57.40.

Lastly, the 78 participants included in the final stage reported minimal exhaustion (N = 58, 52.7%), high levels of cynicism (N = 36, 32.7%), and high professional efficacy (N = 76, 69.1%). The anxiety level rose to high (N = 28, 25.5%), while the depression level remained stable (N = 40, 36.4%). The sleep quality was satisfactory (N = 46, 41.8%), and the mean score of quality of life was 56.82.

The results of the 78 participants who completed all questionnaires at all three timepoints from baseline to 6 months can be found in Table 4 and Appendix A.

Evaluating burnout levels using the ANOVA test for score comparison between the three timepoints indicated a growth of the level of exhaustion, F (2, 154) = 17.007, *p* = 0.001, with the highest level being at 6 months. Similar progressions could be found in the cases of cynicism, F (2, 154) = 21.824, *p* = 0.001, and professional efficacy, F (2, 154) = 11.892, *p* = 0.001.

More than half of the individuals (50.9%) reported mild or moderate anxiety, whereas 27.3% reported significant anxiety at baseline. The ANOVA test results showed stationary anxiety scores between the three timepoints, F (2, 154) = 0.551, *p* = 0.57 (scores between 13 and 13.66).

At baseline, 25.5% reported moderate–severe depression and 20% mild–moderate depression. The total score evolution was also stationary from baseline to 6 months, F(2, 154) = 2.738, *p* = 0.068, with scores between 12, 28 and 13, 84.

A total of 23.6% of the participants reported low-quality sleep at baseline compared to 29.1% at 6 months. The evolution of sleep quality, F (2, 154) = 6.236, *p* = 0.002, showed statistically significant changes between 3 months (lowest scores: 3.8) and 6 months (highest scores: 4.5).

The fear of COVID scale results between baseline and 6 months were also statistically significant, F (2, 154) = 25.701, *p* = 0.001, with lower scores over time.

The results indicate a decrease in the quality of life between the three timepoints reported, F(2, 154) = 3.369, *p* = 0.03, from baseline (58.66) to 6 months (56.82).

According to the linear regression, the quality of life at baseline was influenced by the variables included in the model (F (7, 102) = 44.05, *p* = 0.001). Thus, the predictors for quality of life at baseline were cynicism at baseline (beta = −0.31, t = −2.35, *p* = 0.02), anxiety at baseline (beta = −0.40, t = −2.97, *p* = 0.004), and depression at baseline (beta = −0.50, t = −3.45, *p* = 0.001). These predictors explained 73.4% of the variance in quality of life at baseline (Table 5 and Table 6).

The quality of life at 3 months was influenced by the variables included in the model (F (6, 89) = 100.078, *p* = 0.001). Thus, the predictive factors for quality of life at 3 months were cynicism at 3 months (beta = −0.44, t = −2.79, *p* = 0.006), professional inefficiency at 3 months (beta = 0.46, t = 3.08, *p* = 0.003), and depression at 3 months (beta = −0.82, t = −5.68, *p* = 0.001). These predictors explained 86.2% of the variance in quality of life at 3 months.

At 6 months, the quality of life was influenced by the variables included in the model (F (6, 71) = 93.3r1, *p* = 0.001). Thus, the predictive factors for quality of life at 6 months were cynicism at 6 months (beta = −0.46, t = −2.27, *p* = 0.02), depression at 6 months (beta = −0.94, t = −6.21, *p* = 0.001), and sleep quality at 6 months (beta = 0.21, t = 2.54, *p* = 0.01). These predictors explained 87,8% of the variance in quality of life at 6 months.

## 4. Discussion

In reaching our goal, we established the following objectives, as mentioned in the introduction section: investigate the dynamics of anxiety, depression, burnout, sleep, and quality of life in a group of informal caregivers in the context of pandemic distress, and, subsequently, discover the factors that could influence quality of life during this time.

First and foremost, our results provided a description of the socio-demographic data of the participants, which could also influence their health outcomes. Most of our participants were female (most likely the daughter of the patient), around 55 years old, living in an urban area, married, with children, having a high school or higher education, employed at a job requiring physical presence, and in the best-case scenario, dividing their responsibilities with another two–three caregivers. These findings are consistent with the existing literature. In the United States of America, almost 75% of people with dementia are cared for by informal caregivers, typically their spouses and followed by their daughters. However, compared to 1996, in 2008, male-gender caregivers became more frequent (with an increase of 21%). Moreover, men over 75 years old are more likely to care for their spouse [29]. Current literature suggests that female caregivers have experienced worse mental health during the pandemic compared to male carers, since they are typically expected to look after the household and familial responsibilities; therefore, our results are not surprising since most of the included participants were female [30].

Another important factor contributing to the carer’s burden is the state of the patient. Most of the patients from our study were diagnosed with a mixed form of dementia and had low MMSE scores (mean score = 13), which could explain the increased burden of the carers. According to a recent study from 2022 comparing the burden for different types of dementia, patients with mixed forms were associated with higher burdens of care than patients with Alzheimer’s disease [31].

We also asked participants about their coronavirus status because the data collection period coincided with the COVID-19 pandemic, which had a significant psychological impact that should not be overlooked. Both the caregivers included in the study and the patients had not been diagnosed with COVID, which could explain the low scores in the fear of COVID scale over time. Another explanation for the low scores could be their high levels of education; therefore, the participants had a better understanding of the importance of restrictions. However, more than half of the dementia patients were not vaccinated (58%). Francesco Bruno et al. used multivariable logistic regression analyses in his study, indicating that worries about the possible side effects were significantly related to the vaccination. Interestingly, other studies describe a conspiracy mentality and avoidance of vaccination. Current literature has focused on correlating caregivers’ needs with the pandemic timeline, and most of the studies have found the first 3 months to be the most stressful, with caregivers feeling lonely and isolated, lacking companionship, and having depressive symptoms [32,33].

Moving on to the caregiver burden open-answer questions, many of our participants reported that during COVID, they faced emotional, financial, and physical challenges; some of them described socio-professional difficulties, low accessibility to the healthcare system, and worsening of the patient’s symptoms; therefore, it was no surprise that almost half of them reported financial, physical, and psychological needs. During the COVID-19 pandemic, the healthcare system mobilized rapidly in order to face the challenges of the ongoing crisis, while the patients with ongoing needs and their caregivers felt lost trying to access routine assistance [34,35].

To achieve our first objective, the measurements used showed that the participants’ burnout scores increased on all three levels measured (exhaustion, cynicism, and professional efficacy) from baseline to 6 months, with mild to moderate anxiety at baseline (more than half of the individuals or 50.9%) and moderate–severe depression at baseline (in 25.5% of the participants) and with a decrease in sleep quality throughout this period. Lane N.E. et al. also investigated the prevalence of anxiety and depression in a group of over 600 caregivers during COVID in a cross-sectional study, finding clinically significant anxiety in 28.6% of the participants and depression in 38.8%, having both personal stressors, such as low social support, and caregiving stressors exacerbated by the pandemic [36]. Informal caregivers of older patients with dementia reported high levels of stress, burden, or depression in comparison to carers of patients with other diseases, especially during the pandemic (due to social isolation), having a negative impact on their quality of life [37,38,39,40,41,42,43,44,45,46,47,48].

Regarding our second objective, the results show a decrease in quality of life from baseline (58.66) to 6 months (56.82), influenced by burnout (cynicism and professional efficacy), anxiety, depression, and sleep quality throughout this period. Interestingly, the decline in quality of life when the dread of COVID-19 was decreasing shows that the duration of care increased the caregiver load. These results are similar to the existing literature reporting that the number of hours dedicated to care, the number of chores, and the duration of care are predictive factors influencing quality of life. Other predictors were carer’s age, a spouse as a caregiver, the support received by the caregivers, their experience in care, depressive symptoms, and the carer’s health self-assessment [49].

Previous studies described different aspects of dementia as predictive factors influencing life quality, such as psychological and behavioral symptoms or severity of the disease [50,51]. However, our results showed that anxiety and depressive symptoms, alongside burnout, had a stronger association with the carer’s quality of life. Interestingly, in another study, Takai M. et al. discovered that a combination of depression and burnout symptoms, as well as the patient’s cognitive impairment, best predicted the caregiver’s life quality [52].

One possibility for helping improve caregivers’ quality of life is through telehealth services (electronic platforms for health information with audio/video technology), which have rapidly developed during the pandemic [53,54,55,56,57]. Either synchronous (real-time communication) or asynchronous (previously recorded data) telehealth has rapidly become the primary health delivery system in many countries [58,59].

For caregivers of dementia patients, telehealth consultations could help reduce the costs of travel, lower the risk of infectivity, and grant easy access for the patients living in rural areas [60,61]. However, we should take into consideration the possible disadvantages, such as smartphone ownership among older people (only 61% of people over 65 years own a smartphone). Basically, telehealth can prevent deterioration of cognition in patients living with dementia, relieve caregivers’ distress, provide information for them, and improve their quality of life and their interactions with dementia patients [62,63,64,65,66,67,68].

Several limitations of our study include the following: (1) the small number of participants (110 initial caregivers), thus being insufficient for generalization; (2) telephonic surveys instead of face-to-face evaluations, which, even though they have been widely used during the pandemic, can have issues like connectivity and limitation of question complexity that could be disadvantageous; (3) reliability of survey data—the included participants may not have felt motivated in delivering accurate answers or might have felt uncomfortable answering questions that could portray them negatively.

Among the advantages of our article are the longitudinal structure, allowing us to investigate the variable patterns over time; the statistical interpretation of the data; and the number of participants, considering the coronavirus pandemic restrictions at the time of the data collection.

## 5. Conclusions

A total of 110 caregivers were included in the beginning of the study, most of them female, usually the daughter of the patient, almost 55 years old, living in an urban area, married, with at least one child, with a high school education, employed, and with a job requiring physical presence.

The most frequent challenges encountered by the caregivers were emotional, financial, and physical challenges, leading to changes in all three burnout dimensions, mild–moderate anxiety, moderate–severe depression, low sleep quality, a diminished fear of COVID, and a decrease in quality of life (predicted by burnout, anxiety, depression, and sleep quality).

The longitudinal structure of the study allowed us to detect modifiable variables that influence caregivers’ quality of life over time (burnout, anxiety, depression, sleep quality), therefore creating the base of future studies focusing on interventions for enhancing caregivers’ psychological conceptions.

## Figures and Tables

**Table 1:** Characteristics of the questionnaires used.

Questionnaire	Measure	Scoring
The Maslach Burnout Inventory	Measuring burnout using exhaustion, cynicism, professional efficacy	Exhaustion: 0–18: low burnout level 19–26: moderate ≥27: high Cynicism: 1–5: low level of burnout 6–9: moderate ≥10: high Professional efficacy: ≥40: low level of burnout 34–39: moderate 0–33: high
Hamilton Anxiety Rating Scale (HARS) (clinician administered)	Severity of anxiety using psychic and somatic symptoms	≤17 indicates mild anxiety 18–24: moderate anxiety 25–30: moderate to severe anxiety
Hamilton Depression Rating Scale (HDRS) (clinician administered)	Severity of depression using psychic and somatic symptoms	0–7 is considered normal 8–16: mild depression 17–23: moderate depression ≥24: severe depression
World Health Organization Quality of Life (WHOQOL)	Physical health (seven questions regarding daily activities, mobility, energy, functional capacity, pain, and sleep) Mental health (six questions related to self-image, ability to learn, positive attitudes, negative thoughts, mentality, religion, focus, and mental status) Social relationships (three questions about personal relationships, sex life, social support) Environmental health (eight questions about safety, financial resources, health and social services, the environment in which they live, recreation, opportunities to acquire new knowledge, the environment, and their means of transport	Physical health (raw score range: 7–35) Mental health (raw score range: 6–30) Social relationships (raw score range: 3–15) Environmental health (raw score range: 8–40) Each domain was transformed into a 0–100 scoring algorithm.
Pittsburg Sleep Quality Index (PSQI)	Overall sleep quality (sleep quality, latency, duration, efficiency, sleep disturbances, the use of sleep medication, and dysfunction during the daytime)	A seven-component score was derived from the items, each scored from 0 (no difficulty) to 3 (severe difficulty). The component scores were summed, ranging from 0 to 21.
Fear of COVID-19 scale	Fear of COVID-19	Total score range of 7–35
Caregiver burden scale	Burden of the caregiver (transportation, housekeeping, cooking, shopping, decision making, financial record keeping, walking, making house repairs, farming/yardwork, administering medication, dressing, bathing, eating, toileting, leaving patient unattended)	Total score range of 1–15

**Table 2:** Socio-demographic data of the included informal caregivers at baseline.

	Mean	Standard Deviation
**Age**	55.22	55.22
	**Frequency**	**Percent**
**Gender**		
Female	78	70.9
Male	32	29.1
**Living area**		
Urban	88	80.0
Rural	22	20.0
**Marital status**		
Single	6	5.5
Married	76	69.1
Divorced	12	10.9
Widow	10	9.1
In a relationship	6	5.5
**Education**		
Middle school	6	5.5
High school	58	52.7
University	46	41.8
**Occupation**		
Student	2	1.8
Working	68	61.8
Unemployed	4	3.6
Retired	36	32.7
**Working status**		
Physical presence	48	43.6
Online presence	6	5.5
Hybrid presence	18	16.4

**Table 3:** Questions regarding the patient with dementia.

Relationship to the Patient	Frequency	Percent
**Nephew/niece**	14	12.7
**Wife/husband**	20	18.2
**Daughter/son**	68	61.8
**Daughter-in-law**	8	7.3
**Type of dementia**		
**Atypical or mixed Alzheimer’s dementia**	64	58.2
**Alzheimer’s dementia, late onset**	12	10.9
**Alzheimer’s dementia, unspecified**	28	25.5
**Alzheimer’s dementia, early onset**	6	5.5
	**Mean**	**Standard deviation**
**Last MMSE score**	13.32	6.789
**Total number of caregivers for one patient**	1.67	0.692
**Caregiver burden**	11.05	3.270

**Table 4:** Mean scores of the questionnaires used at all three timepoints.

Measure	Baseline		3 Months		6 Months		*p*
	Mean	Std. Error	Mean	Std. Error	Mean	Std. Error	
Maslach Burnout Inventory							
Emotional exhaustion	7.33	0.667	9.28	0.764	11.36	0.962	0.001
Cynicism	5.308	0.627	7.179	0.732	9.923	0.958	0.001
Professional efficacy	7.282	0.890	8.949	0.969	11.641	1.256	0.001
HARS	13.000	1.286	13.154	1.232	13.667	1.217	0.57
HDRS	12.282	1.235	13.282	1.184	13.846	1.203	0.068
Pittsburg Sleep Quality Index	4.205	0.356	3.769	0.351	4.513	0.365	0.002
Fear of COVID-19	10.795	0.586	8.179	0.263	8.179	0.342	0.001
WHOQOL-BREF 26	58.667	1.354	57.359	1.316	56.821	1.196	0.03

**Table 5:** Correlations with quality of life.

Measure	Baseline			3 Months			6 Months		
	r	*p*	Correlation	r	*p*	Correlation	r	*p*	Correlation
Maslach Burnout Inventory									
Emotional exhaustion	−0.66	0.001	Inverse and moderate	-	-	-	-	-	-
Cynicism	−0.64	0.001	Inverse and moderate	−0.76	0.001	Inverse and strong	−0.75	0.001	Inverse and strong
Professional efficacy	−0.65	0.001	Inverse and moderate	−0.75	0.001	Inverse and strong	−0.80	0.001	Inverse and strong
HARS	−0.83	0.001	Inverse and strong	−0.88	0.001	Inverse and strong	−0.89	0.001	Inverse and strong
HDRS	−0.84	0.001	Inverse and strong	−0.92	0.001	Inverse and strong	−0.93	0.001	Inverse and strong
Pittsburg Sleep Quality Index	−0.71	0.001	Inverse and strong	−0.70	0.001	Inverse and strong	−0.73	0.001	Inverse and strong
Fear of COVID-19	−0.36	0.001	Inverse and low	−0.42	0.001	Inverse and moderate	−0.63	0.001	Inverse and moderate

**Table 6:** Overview of the models that predicted changes in life quality.

Timepoint	Model	R	R Square	Adjusted R Square	Std. Error of the Estimate	Change Statistics	
						R Square Change	F Change
Baseline	(Constant), professional efficacy, cynicism, exhaustion, anxiety, depression, caregiver burden, sleep quality	0.867	0.751	0.734	5.614	0.000	0.003
3 months	(Constant), professional efficacy, cynicism, exhaustion, anxiety, depression	0.933	0.871	0.862	4.300	0.003	2.264
6 months	(Constant), professional efficacy, cynicism, exhaustion, anxiety, depression, sleep quality	0.942	0.887	0.878	3.691	0.010	6.466

**Figure A1:**
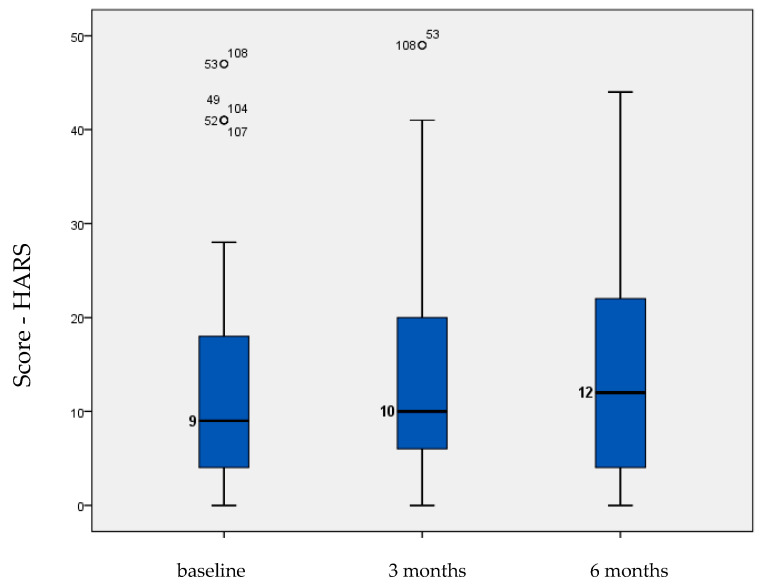
HARS (Hamilton Anxiety Rating Scale) score comparison between the 3 timepoints.

**Figure A2:**
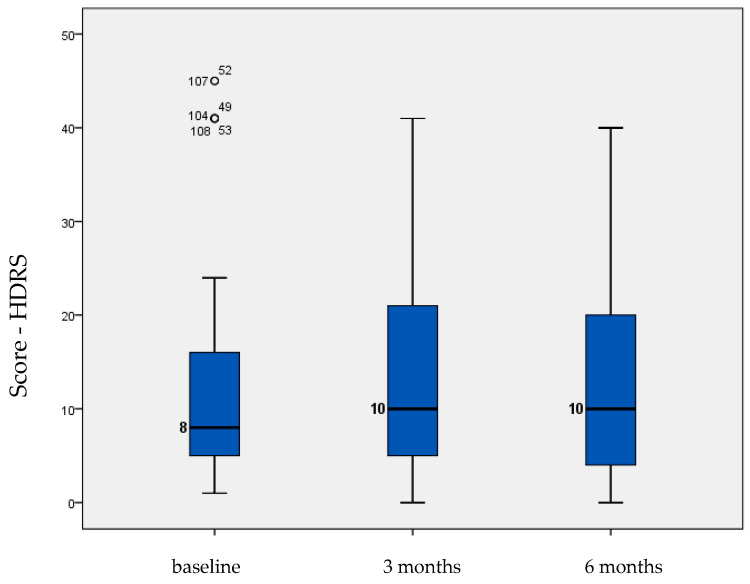
HDRS (Hamilton Depression Rating Scale) score comparison between the 3 timepoints.

**Figure A3:**
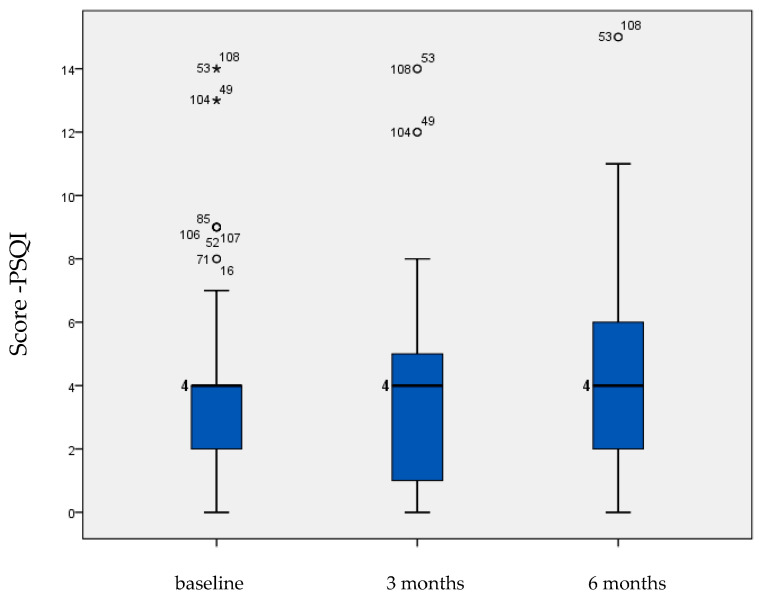
PSQI (Pittsburg Sleep Quality Index) score comparison between the 3 timepoints.

**Figure A4:**
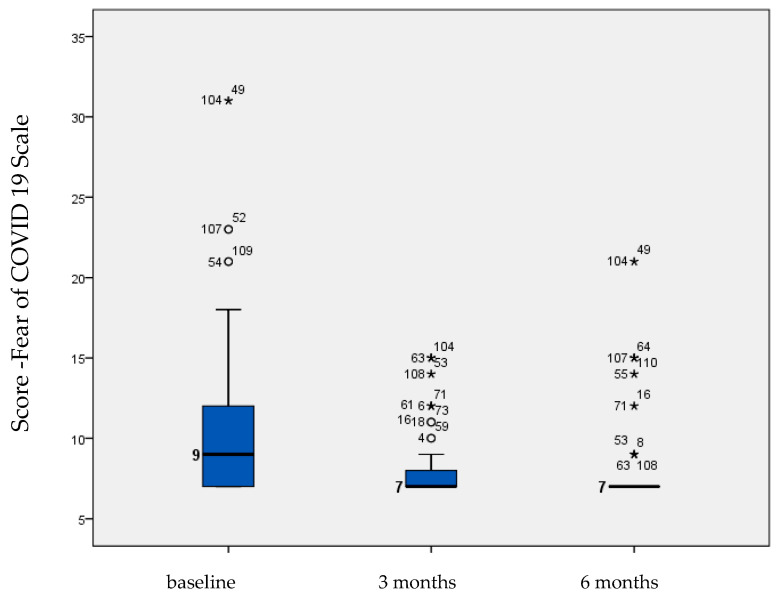
Fear of COVID-19 score comparison between the 3 timepoints.

## Data Availability

All data reported in the article are available in anonymized form upon request.

## References

[1] Alzheimer’s Association Report (2020). Alzheimer’s Disease Facts and Figures. Alzheimers Dement..

[2] Onetiu V., Sorina Maria Aurelian A., Capisizu F., Ileana Codruta Zus K. (2016). Cost of Dementia in Romania: A Cross-Sectional Cost-of-Illness Study Undertaken in Bucharest. Zdr. Pu-Bliczne I Zarządzanie.

[3] Cheng S.-T. (2017). Dementia Caregiver Burden: A Research Update and Critical Analysis. Curr. Psychiatry Rep..

[4] Archbold P.G. (1983). Impact of Parent Caring on Women. Proceedings of the XII International Congress of Gerontology.

[5] Rapp S.R., Chao D. (2000). Appraisals of Strain and of Gain: Effects on Psychological Wellbeing of Caregivers of Dementia Patients. Aging Ment. Health.

[6] Jeste D.V., Mausbach B., Lee E.E. (2021). Caring for Caregivers/Care Partners of Persons with Dementia. Int. Psychogeriatry.

[7] Lowery K., Mynt P., Aisbett J., Dixon T., Obrien J., Ballard C. (2000). Depression in the Carers of Dementia Sufferers: A Com-Parison of the Carers of Patients Suffering from Dementia with Lewy Bodies and the Carers of Patients with Alzheimer’s Disease. J. Affect. Disord..

[8] Brodaty H., Hadzi-Pavlovic D. (1990). Psychosocial Effects on Carers of Living with Persons with Dementia. Aust. N. Z. J. Psychiatry.

[9] Cohen G., Russo M.J., Campos J.A., Allegri R.F. (2020). Living with Dementia: Increased Level of Caregiver Stress in Times of COVID-19. Int. Psychogeriatry.

[10] Aledeh M., Habib Adam P. (2020). Caring for Dementia Caregivers in Times of the COVID-19 Crisis: A Systematic Review. Am. J. Nurs. Res..

[11] Bailey C., Guo P., MacArtney J., Finucane A., Swan S., Meade R., Wagstaff E. (2022). The Experiences of Informal Carers during the COVID-19 Pandemic: A Qualitative Systematic Review. Int. J. Environ. Res. Public Health.

[12] Eggert S., Teubner C., Budnick A., Gellert P., Kuhlmey A. (2020). Pflegende Angehörige in Der COVID-19-Krise.

[13] Wolf-Ostermann K., Schmidt A., Preuß B., Heinze F., Seibert K., Friedrich A.-C., Domhoff D., Stolle C., Rothgang H. (2020). Pflege in Zeiten von Corona: Ergebnisse Einer Deutschlandweiten Querschnittbefragung von Ambulanten Pflegediensten Und Teilstationären Einrichtungen. Pflege.

[14] Leiblfinger M., Prieler V., Schwiter K., Steiner J., Benazha A., Lutz H. (2020). Impact of COVID-19 Policy Responses on Live-in Care Workers in Austria, Germany, and Switzerland. J. Long Term Care.

[15] Rothgang H., Wolf-Ostermann K., Domhoff D., Friedrich A.-C., Heinze F., Heß M. (2020). Zur Situation Der Häuslichen Pflege in Deutschland Während Der Corona-Pandemie-Ergebnisse Einer Online—Befragung von Informellen Pflegepersonen Im Erwerbsfähigen Alter.

[16] Bergmann M., Wagner M. (2021). The Impact of COVID-19 on Informal Caregiving and Care Receiving across Europe during the First Phase of the Pandemic. Front. Public Health.

[17] Ciobanu A.M., Damian A.C., Neagu C. (2021). Association between Burnout and Immunological and Endocrine Alterations. Rom. J. Morphol. Embryol..

[18] Enciu B.G., Tănase A.A., Drăgănescu A.C., Aramă V., Pițigoi D., Crăciun M.-D. (2022). The COVID-19 Pandemic in Romania: A Comparative Description with Its Border Countries. Healthcare.

[19] Folstein M.F., Folstein S.E., McHugh P.R. (1975). Mini-Mental State. J. Psychiatry Res..

[20] Portoghese I., Leiter M.P., Maslach C., Galletta M., Porru F., D’Aloja E., Finco G., Campagna M. (2018). Measuring Burnout among University Students: Factorial Validity, Invariance, and Latent Profiles of the Italian Version of the Maslach Burnout Inventory Student Survey (MBI-SS). Front. Psychol..

[21] Hallit S., Haddad C., Hallit R., Akel M., Obeid S., Haddad G., Soufia M., Khansa W., Khoury R., Kheir N.P. (2020). Validation of the Hamilton anxiety rating scale and state trait anxiety inventory A and B in Arabic among the Lebanese population. Clin. Epidemiol. Glob. Health.

[22] Wickramasinghe N.D., Dissanayake D.S., Abeywardena G.S. (2018). Validity and Reliability of the Maslach Burnout Inventory-Student Survey in Sri Lanka. BMC Psychol..

[23] Olden M., Rosenfeld B., Pessin H., Breitbart W. (2009). Measuring Depression at the End of Life: Is the Hamilton Depression Rating Scale a Valid Instrument?. Assessment.

[24] Ilić I., Šipetić-Grujičić S., Grujičić J., Živanović Mačužić I., Kocić S., Ilić M. (2019). Psychometric Properties of the World Health Organization’s Quality of Life (WHOQOL-BREF) Questionnaire in Medical Students. Medicina.

[25] Almarabheh A., Ghamdi M.A., Elbarbary A., Alqashar A., Alserdieh F., Alahmed F., Alhaddar H., AlSada L., Yosri M., Omran M. (2021). Validity and Reliability of the WHOQOL-BREF in the Measurement of the Quality of Life of Sickle Disease Patients in Bahrain. Res. Sq..

[26] Midorikawa H., Aiba M., Lebowitz A., Taguchi T., Shiratori Y., Ogawa T., Takahashi A., Takahashi S., Nemoto K., Arai T. (2021). Confirming Validity of The Fear of COVID-19 Scale in Japanese with a Nationwide Large-Scale Sample. PLoS ONE.

[27] Ahorsu D.K., Lin C.-Y., Imani V., Saffari M., Griffiths M.D., Pakpour A.H. (2022). The Fear of COVID-19 Scale: Development and Initial Validation. Int. J. Ment. Health Addict..

[28] Wang L., Wu Y.-X., Lin Y.-Q., Wang L., Zeng Z.-N., Xie X.-L., Chen Q.-Y., Wei S.-C. (2022). Reliability and Validity of the Pittsburgh Sleep Quality Index among Frontline COVID-19 Health Care Workers Using Classical Test Theory and Item Response Theory. J. Clin. Sleep Med..

[29] Brodaty H., Donkin M. (2009). Family Caregivers of People with Dementia. Dialogues Clin. Neurosci..

[30] Wister A., Li L., Mitchell B., Wolfson C., McMillan J., Griffith L.E., Kirkland S., Raina P., Costa A., Anderson L. (2022). Levels of Depression and Anxiety among Informal Caregivers during the COVID-19 Pandemic: A Study Based on the Canadian Longitudinal Study on Aging. J. Gerontol. B Psychol. Sci. Soc. Sci..

[31] Huang W.-C., Chang M.-C., Wang W.-F., Jhang K.-M. (2022). A Comparison of Caregiver Burden for Different Types of Dementia: An 18-Month Retrospective Cohort Study. Front. Psychol..

[32] Bruno F., Malvaso A., Chiesi F., Laganà V., Servidio R., Isella V., Ferrarese C., Gottardi F., Stella E., Agosta F. (2022). COVID-19 Vaccine Uptake among Family Caregivers of People with Dementia: The Role of Attitudes toward Vaccination, Perceived Social Support and Personality Traits. Front. Psychol..

[33] Oortwijn R., Van Leeuwen F., Ren D. How Openness to Experience Relates to Conspiracy Mentality and Vaccine Hesitancy. http://arno.uvt.nl/show.cgi?fid=151204.

[34] Le Couteur D.G., Anderson R.M., Newman A.B. (2020). COVID-19 through the Lens of Gerontology. J. Gerontol. A Biol. Sci. Med. Sci..

[35] Rimmer A. (2020). COVID-19: GPs Can Stop Health Checks for over 75s and Routine Medicine Reviews. BMJ.

[36] Lane N.E., Hoben M., Amuah J.E., Hogan D.B., Baumbusch J., Gruneir A., Chamberlain S.A., Griffith L.E., McGrail K.M., Corbett K. (2022). Prevalence and Correlates of Anxiety and Depression in Caregivers to Assisted Living Residents during COVID-19: A Cross-Sectional Study. BMC Geriatr..

[37] Dourado M.C.N., Belfort T., Monteiro A., de Lucena A.T., Lacerda I.B., Gaigher J., Baptista M.A.T., Brandt M., Kimura N.R., de Souza N. (2020). COVID-19: Challenges for Dementia Care and Research. Dement. Neuropsychol..

[38] Archer J., Reiboldt W., Claver M., Fay J. (2021). Caregiving in Quarantine: Evaluating the Impact of the Covid-19 Pandemic on Adult Child Informal Caregivers of a Parent. Gerontol. Geriatr. Med..

[39] Budnick A., Hering C., Eggert S., Teubner C., Suhr R., Kuhlmey A., Gellert P. (2021). Informal Caregivers during the COVID-19 Pandemic Perceive Additional Burden: Findings from an Ad-Hoc Survey in Germany. BMC Health Serv. Res..

[40] Cohen S.A., Kunicki Z.J., Drohan M.M., Greaney M.L. (2021). Exploring Changes in Caregiver Burden and Caregiving Intensity Due to COVID-19. Gerontol. Geriatr. Med..

[41] Zwaanswijk M., Peeters J.M., van Beek A.P.A., Meerveld J.H.C.M., Francke A.L. (2013). Informal Caregivers of People with Dementia: Problems, Needs and Support in the Initial Stage and in Subsequent Stages of Dementia: A Questionnaire Survey. Open Nurs. J..

[42] Giebel C., Lord K., Cooper C., Shenton J., Cannon J., Pulford D., Shaw L., Gaughan A., Tetlow H., Butchard S. (2021). A UK Survey of COVID-19 Related Social Support Closures and Their Effects on Older People, People with Dementia, and Carers. Int. J. Geriatr. Psychiatry.

[43] Park S.S. (2021). Caregivers’ Mental Health and Somatic Symptoms during COVID-19. J. Gerontol. B Psychol. Sci. Soc. Sci..

[44] Savla J., Roberto K.A., Blieszner R., McCann B.R., Hoyt E., Knight A.L. (2021). Dementia Caregiving during the “Stay-at-Home” Phase of COVID-19 Pandemic. J. Gerontol. B Psychol. Sci. Soc. Sci..

[45] Sheth K., Lorig K., Stewart A., Parodi J.F., Ritter P.L. (2021). Effects of COVID-19 on Informal Caregivers and the Development and Validation of a Scale in English and Spanish to Measure the Impact of COVID-19 on Caregivers. J. Appl. Gerontol..

[46] Bussè C., Barnini T., Zucca M., Rainero I., Mozzetta S., Zangrossi A., Cagnin A. (2022). Depression, Anxiety and Sleep Alterations in Caregivers of Persons with Dementia after 1-Year of COVID-19 Pandemic. Front. Psychiatry.

[47] Daley S., Farina N., Hughes L., Armsby E., Akarsu N., Pooley J., Towson G., Feeney Y., Tabet N., Fine B. (2022). COVID-19 and the Quality of Life of People with Dementia and Their Carers—The TFD-C19 Study. PLoS ONE.

[48] Leggett A.N., Carmichael A., Leonard N., Jackson J., Kirch M., Solway E., Kullgren J.T., Singer D., Malani P.N., Gonzalez R. (2021). Care Challenges Due to COVID-19 and Mental Health among Caregivers of U.S. Adults with a Chronic or Disabling Condition. Innov. Aging.

[49] Ślusarska B., Bartoszek A., Kocka K., Deluga A., Chrzan-Rodak A., Nowicki G. (2019). Quality of Life Predictors in Informal Caregivers of Seniors with a Functional Performance Defici—An Example of Home Care in Poland. Clin. Interv. Aging.

[50] Ichimiya A., Igata R., Ogomori K. (2001). Care-Givers IT. QOL and the Burden of Care-Giving for De-Mented Elderly at Home. Ronen Seishin Igaku Zasshi.

[51] Glozman J.M. (2004). Quality of Life of Caregivers. Neuropsychol. Rev..

[52] Takai M., Takahashi M., Iwamitsu Y., Oishi S., Miyaoka H. (2011). Subjective Experiences of Family Caregivers of Patients with Dementia as Predictive Factors of Quality of Life: Predictive Factors for QOL in Caregivers. Psychogeriatrics.

[53] Hajjar L., Kragen B. (2021). Timely Communication through Telehealth: Added Value for a Caregiver during COVID-19. Front. Public Health.

[54] Roach P., Zwiers A., Cox E., Fischer K., Charlton A., Josephson C.B., Patten S.B., Seitz D., Ismail Z., Smith E.E. (2021). Understanding the Impact of the COVID-19 Pandemic on Well-Being and Virtual Care for People Living with Dementia and Care Partners Living in the Community. Dementia.

[55] Cuffaro L., Lorenzo D., Bonavita F. (2020). Dementia Care and COVID-19 Pandemic: A Necessary Digital Revolu-Tion. Neurol. Sci.

[56] O’Connor M.K., Nicholson R., Epstein C., Donley T., Salant R., Nguyen A.H., Shirk S., Stevenson E., Mittelman M.S. (2023). Telehealth Support for Dementia Caregivers during the COVID-19 Pandemic: Lessons Learned from the NYU Family Support Program. Am. J. Geriatr. Psychiatry.

[57] Quail Z., Bolton L., Massey K. (2021). Digital Delivery of Non-Pharmacological Intervention Programmes for People Living with Dementia during the COVID-19 Pandemic. BMJ Case Rep..

[58] Beheshti L., Kalankesh L.R., Doshmangir L., Farahbakhsh M. (2022). Telehealth in Primary Health Care: A Scoping Review of the Literature. Perspect. Health Inf. Manag..

[59] Rajasekaran K. (2020). Access to Telemedicine-Are We Doing All That We Can during the COVID-19 Pandemic?. Otolaryngol Head Neck Surg..

[60] Dang S., Gomez-Orozco C.A., van Zuilen M.H., Levis S. (2018). Providing Dementia Consultations to Veterans Using Clinical Video Telehealth: Results from a Clinical Demonstration Project. Telemed. J. E-Health.

[61] Gately M.E., Trudeau S.A., Moo L.R. (2019). In-Home Video Telehealth for Dementia Management: Implications for Rehabilitation. Curr. Geriatr. Rep..

[62] Langford A.T., Solid C.A., Scott E., Lad M., Maayan E., Williams S.K., Seixas A.A. (2019). Mobile Phone Ownership, Health Apps, and Tablet Use in US Adults with a Self-Reported History of Hypertension: Cross-Sectional Study. JMIR MHealth UHealth.

[63] Panerai S., Raggi A., Tasca D., Musso S., Gelardi D., Prestianni G., Catania V., Muratore S., Ferri R. (2021). Telephone-Based Reality Orientation Therapy for Patients with Dementia: A Pilot Study during the COVID-19 Outbreak. Am. J. Occup. Ther..

[64] Lai F.H.-Y., Yan E.W.-H., Yu K.K.-Y., Tsui W.-S., Chan D.T.-H., Yee B.K. (2020). The Protective Impact of Telemedicine on Persons with Dementia and Their Caregivers during the COVID-19 Pandemic. Am. J. Geriatr. Psychiatry.

[65] Capozzo R., Zoccolella S., Frisullo M., Barone R., Dellabate M., Barulli M. (2020). Telemedicine for Delivery of Care in Fron-Totemporal Lobar Degeneration during COVID-19 Pandemic: Results from Southern Italy. J. Alzheimer’s Dis..

[66] Cooper C., Mansour H., Carter C., Rapaport P., Morgan-Trimmer S., Marchant N.L., Poppe M., Higgs P., Brierley J., Solomon N. (2021). Social Connectedness and Dementia Prevention: Pilot of the APPLE-Tree Video-Call Intervention during the COVID-19 Pandemic. Dementia.

[67] Masoud S.S., Meyer K.N., Sweet M., Prado L., White P.J. (2021). We Don’t Feel so Alone: A Qualitative Study of Virtual Memory Cafés to Support Social Connectedness among Individuals Living with Dementia and Care Partners during COVID-19. Front Public Health.

[68] Gedde M.H., Husebo B.S., Erdal A., Puaschitz N.G., Vislapuu M., Angeles R.C., Berge L.I. (2021). Access to and Interest in Assistive Technology for Home-Dwelling People with Dementia during the COVID-19 Pandemic (Pan.deM). Int. Rev. Psychiatry.

